# The Zipperator! A Novel Model to Simulate Penile Zipper Entrapment

**DOI:** 10.21980/J8NS8F

**Published:** 2021-10-15

**Authors:** Dale Till, Simran Ghuman, Luke Kim, Ryan Roleson, Jessica Morrison, Sage Wexner

**Affiliations:** * University of California Davis, Department of Emergency Medicine, Davis, CA; ^ University of California San Francisco School of Medicine, San Francisco, CA; ‡ Kern Medical Center, Department of Emergency Medicine, Bakersfield, CA; ** University of Pittsburgh Medical Center, Department of Emergency Medicine, Pittsburgh, PA; ^^ Kern Medical Center, Simulation Center, Bakersfield, CA

## Abstract

**Audience:**

The Zipperator training model is designed for emergency medicine resident physicians and physicians.

**Introduction:**

Zipper entrapment injuries are an uncommon cause of penile injury in the emergency department, representing an incidence of less than 0.5% of pediatric emergency department visits.^1^ However, they are one of the most common causes of genital injuries in young boys.^1^,^2^ Various methods proposed for releasing the entrapped tissue range from the use of mineral oil as lubrication to techniques to release the zipper mechanism or, in extreme cases, surgical procedures.^3^–^6^ A well-designed simulated task trainer would allow learners to practice these methods in a controlled environment conducive to learning 7,8. Given the low frequency of the chief complaint and with a wide variety of release techniques available, the purpose of this study was to build a simulation model that could improve learner confidence in troubleshooting this rarely performed procedure. Although past studies have designed similar task trainers, this novel model was built using a low-cost device (“Operation”) to provide real-time alarm noises that reasonably simulate distress and procedural anxiety for both the patient and provider. Although pain and anxiety are separate outcomes from zipper entrapment release, including a component of these emotions may mimic the same emotional states that patients, their parental unit, and perhaps their provider may experience regarding this chief complaint given its rarity, anatomic vulnerability, and the overall sensitivity of the complaint’s nature.

**Educational Objectives:**

After training on the Zipperator, learners will be able to:

1. Demonstrate at least two techniques for zipper release and describe how methods would extrapolate to a real patient.
2. Verbalize increased comfort with the diagnosis of zipper entrapment.
3. Present a plan of care for this low-volume, high-anxiety presentation.

**Educational Methods:**

As part of a voluntary Emergency Medicine curriculum at two different sites, we constructed an inexpensive model for penile zipper entrapment using a household gameboard, “Operation,” and materials that are easily obtainable and assembled in any emergency department. “Operation” was selected for its ability to produce alarm noises in response to excessive movement, which would reasonably simulate distress and procedural anxiety that may be experienced by both the patient and provider. This task trainer was used to teach medical students and post graduate year (PGY) 1–4 resident learners. A brief hypothetical situation was presented to learners, highlighting patient and paternal unit anxiety. Following this, learners were given a survey and asked to complete pre-model training questions immediately prior to using the simulated model. Learners were then given the opportunity for hands-on skills-based practice. Postmodel training questions were made available in the same survey immediately following the exercise.

**Research Methods:**

This exercise was offered at two sites over a two-year period. Sixty learners participated in the exercise. Participation was voluntary, was not graded nor shared with the residency director, and all feedback was formative in nature. Selected faculty and research assistants provided asynchronous opportunities for learners to practice on the model. Before the exercise, the faculty or research assistant on site presented a brief hypothetical situation to simulate patient and paternal unit anxiety that could be expected in this chief complaint. Each learner was then allowed to select a variety of tools and methods to practice zipper entrapment release. Learners were asked to begin a survey prior to training on the model, and then complete the survey immediately after training on the model to evaluate its educational value. The survey created for this study consisted of a structured questionnaire that contained close-ended questions. Measures evaluated include experience with prior zipper entrapments, comfort with zipper entrapments before and after training on the simulated model, and user experience.

**Results:**

Before the exercise, 68.3% of learners described their comfort with managing future zipper entrapments as very uncomfortable or totally uncomfortable. Although only 8.3% of learners had treated the zipper entrapment complaint prior to the exercise, 100% of those who had experienced treating the complaint felt that the simulated model was at least somewhat reflective of their experience with a real patient. 71.7% of the learners found the experience enjoyable, although 20% found the experience totally unenjoyable, of note, for unclear reasons and with unclear significance or etiology. After the exercise, 71.7% of learners indicated they felt comfortable to very comfortable regarding future cases of zipper entrapment.

**Discussion:**

Through the use of a well-known household board game and supplies commonly found in the emergency department, we created a simulated model that could be easily replicated. This simple model provided practice of the hand motions necessary for zipper entrapment release, as well as familiarity with the mental and physical approaches to dislodging the entrapment. The resident physicians who had had a prior zipper entrapment patient reported the model was somewhat similar to actual patient encounters. Overall, this model was well-received by the learners, with most expressing it was enjoyable and feeling it increased their confidence for treating this chief complaint. Some learners had noted the experience was totally unenjoyable. This measure may not be an appropriate endpoint, however, and incongruencies may be addressed by implementing prizes or friendly competition for enjoyment. Another limitation of this study is the leap taken between movement and patient comfort. While possible that learners can manipulate the model to reduce movement of the needle without meaningful reduction in zipper movement, observation by the instructor was sufficient to ensure this finding was not observed in our learner population. We therefore submit this cheap, simple model as a potential method to teach approaches to teaching a low frequency, high anxiety chief complaint.

**Topics:**

Penile entrapment model, penile entrapment release, Emergency Medicine, Urology, Clinical/Procedural Skills Training.

## USER GUIDE

**Table:** 

List of Resources:	
Abstract	1
User Guide	4

**Learner Audience:**

Medical Students, Interns, Junior Residents, Senior Residents

**Time Required for Implementation:**

Approximately 10 minutes will be needed to assemble the Zipperator. Although learners were not specifically timed, most learners spent an average of 5 to 20 minutes exploring the different techniques of the task trainer, including some who wanted to practice more than once. Therefore, it is recommended that learners dedicate at least 20 minutes to the exercise and up to 40 minutes for those who would like extra practice. One session with the task trainer is sufficient for learners to report comfort with the procedure.

**Recommended Number of Learners per Instructor:**

Due to brevity of each session, we recommend a ratio of 1 model for every 1 or 2 learners with a faculty or research assistant ratio of 1:1 or 1:2 for a similar experience. However, given the flexibility of this task trainer, other ratios could be explored.

**Topics:**

Penile entrapment model, penile entrapment release, Emergency Medicine, Urology, Clinical/Procedural Skills Training.

**Objectives:**

After training on the model, the learners will be able to:

1. Demonstrate at least 2 techniques for zipper release and describe how methods would extrapolate to a real patient.
2. Verbalize increased comfort with the diagnosis of zipper entrapment.
3. Present a plan of care for this low-volume, highanxiety presentation.

### Linked objectives and methods

The Operation game used in the Zipperator was selected for its alarm feature, which simulates the pain and distress of the simulated pediatric patient. This set-up would encourage learners to explore and attempt various zipper entrapment release techniques with real-time feedback through the alarm feature (objective 1). Hands-on training with the model followed by a debrief will increase learner confidence and comfort with clinical decision-making for this rarely performed procedure (objectives 2, and 3).

Simulated task trainers provide a controlled, safe environment for learners to practice essential procedural skills without risk to a live patient.^7^,^8^ Additionally, past studies indicate that simulation can play a vital role in improving learner confidence and competence as well as patient safety.^7^–^9^ Thus, using simulation-based medical education is well-suited to the low frequency, high anxiety presentation of zipper entrapment injuries.

### Recommended pre-reading for instructor

- Bothner J. Management of zipper entrapment injuries. In: Post T, ed. UpToDate. Waltham, Mass.: UpToDate; 2021. www.uptodate.com. Accessed Sept. 23, 2021. At: https://www.uptodate.com/contents/management-of-zipper-entrapment-injuries/
- Catalano AW, Amii RN. Chapter 183: Zipper Injury Management. In: Reichman EF, ed. *Reichman’s Emergency Medicine Procedures*. 3^rd^ ed. 2018:1519–1523.
- Davis JE. Chapter 93: Male Genital Problems. In: Tintinalli JE, Ma OJ, Yealy DM, et al, eds. *Tintinalli’s Emergency Medicine: A Comprehensive Study Guide*. 9^th^ Ed. McGraw-Hill; 2020:594.
- McCollough M, Rose E. Chapter 173: Renal and Genitourinary Tract Disorders. In: Walls RM, Hockberger RS, Gauschae-Hill M, eds. *Rosen’s Emergency Medicine*. 9^th^ Ed. Elsevier. 2018:2168.

### Learner responsible content (LRC)

Learner responsible content is not indicated prior to attending the exercise, especially when used for “just in time” training.

### Associated content

- Bothner J. Management of zipper entrapment injuries. In: Post T, ed. UpToDate. Waltham, Mass.: UpToDate; 2021. www.uptodate.com. Accessed Sept. 23, 2021. At: https://www.uptodate.com/contents/management-of-zipper-entrapment-injuries/
- https://wikem.org/wiki/Zipper_injury_to_penis
- https://wiki2.org/en/Penile_injury

### Implementation Methods

The Zipperator requires assembly prior to the exercise. All necessary tools should be laid out for easy access. We recommend the instructor spends approximately 10–15 minutes reviewing current literature on zipper entrapment release. During the didactic session, a brief knowledge assessment and familiarity with diagnosis and techniques can be performed. Following this introduction, learners should explore the model and various tools available to practice this procedure. After a few minutes, the instructor can provide feedback and offer some suggested techniques to guide the learner. Upon completion of the exercise, the instructor can lead a debrief on learner experience with the tools provided and a reminder of current practice recommendations.

### List of items required to replicate this innovation

Many of the recommended materials may be found around the ER, usually discarded from kits. Item 8 (Dremel) specifically should be available in most emergency rooms through engineering.

Items required to build the task trainer include:

1. Classic Operation Game (https://www.target.com/p/operation-board-game/-/A-10855290)
2. Trauma shears
3. Roll of tape
4. #18g needle

A list of tools that can be used for zipper entrapment release includes:

1. Trauma shears
2. Screwdriver
3. Pliers
4. Wire cutters
5. Dremel

### Approximate cost of items to create this innovation

The model can be created using a common household game which costs less than $20.00 and materials that are easily obtainable in an emergency department, also for under $20.00.

### Detailed methods to construct this innovation

1. Start with the Hasbro game, “Operation,” as the base for the model.
2. Affix the game probe to a roll of tape using an #18g needle (such as used for medication administration). The #18g needle should protrude approximately one half-inch below the tape roll edge to allow it to sit inside the wells built into the game.
3. Test the electrical circuit by manipulating the needle so that it provides metal-to-metal electrical contact with the game board. A vibration and illumination of the red light on the “patient’s” nose will occur if the electrical circuit is complete.
4. Obtain a nitrile glove, such as that found in a foley catheter or sterile glove kit. This will be used to simulate the penile skin.
5. Thread the game probe through the nitrile glove.
6. Entrap the nitrile glove in the zipper using a single swift motion with a portion of the glove held between the two sides of the zipper.
7. Place this glove-zipper apparatus on the operation game with the probe hanging in one of the wells.
8. Test the game to see if movements greater than 2–4mm cause the needle to touch the wall of the game. Metal-to-metal contact between the needle and wall of the game should produce a vibration and illumination of the red light on the “patient’s” nose.

**Figure:**
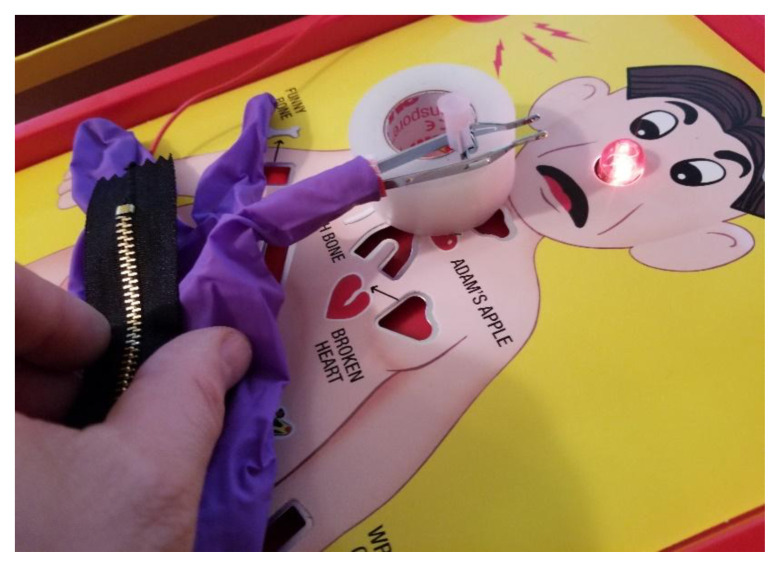
Zipperator! complete model set-up: Author’s own image

A full set up of this task trainer may be viewed on YouTube via the following link: https://youtu.be/5hRikgqKLJY

### Results and tips for successful implementation

This model has been used as a quick teaching point. This model could additionally be used for “just in time” teaching. For instance, in the event of a patient presenting with zipper entrapment, this model could be used to review the various techniques with the alarm serving as a reminder to provide analgesia or anxiolysis. Typical with the rarity of this chief complaint, no patients checked in with this chief complaint during the course of the educational endeavor, and therefore, “just in time teaching” was not used in this study. We implemented this training during our brief teaching sessions between shifts, or before or after sign-out. Sixty total volunteers trained with this model, with over half the learners being emergency medicine residents with one to four years of training. Results were measured through an online survey before and after training on the model; responses were measured using a 7-point Likert scale (1=totally comfortable and 7=totally uncomfortable). Before the exercise, 68.3% of the learners described their comfort with future zipper entrapments as uncomfortable or very uncomfortable. Only 8.3% had treated the zipper entrapment complaint prior to the exercise. Of those, 100% felt that the model was somewhat reflective of their experience with a real patient. 71.7% of the learners found the experience enjoyable, although of note, 20% found the experience totally unenjoyable. After the exercise almost half of the learners, 41.7%, indicated they felt completely or very comfortable regarding future cases of zipper entrapment. No modifications were made as a result of the implementation.

## Supplementary Information


